# Factors hindering effective uptake of medical male circumcision at Untunjambili area in KwaZulu-Natal, South Africa

**DOI:** 10.4102/hsag.v24i0.1305

**Published:** 2019-10-10

**Authors:** Promise N. Sangweni, Thandisizwe R. Mavundla, Pule S. Moabi

**Affiliations:** 1 Department of Health Studies, University of South Africa, Pretoria, South Africa; 2 Clinical Teaching Department, Scott College of Nursing, Morija, South Africa

**Keywords:** factor, influence, uptake, circumcision, medical male circumcision, men

## Abstract

**Background:**

Before 19th century, in KwaZulu-Natal, South Africa, male circumcision was regarded as a right to passage to manhood; however, during the ruling of King Shaka Zulu, circumcision was abolished. It was only in 2010 that it was re-introduced, but this time in the form of medical male circumcision. The aim was to circumcise as many men as possible to avert new human immunodeficiency virus (HIV) infections, but few males utilise the circumcision services.

**Aim:**

The aim of this study was to gain an understanding of the factors that hinder effective uptake of medical male circumcision.

**Setting:**

This study was conducted at Untunjambili area under iLembe district in KwaZulu-Natal province.

**Methods:**

This qualitative, exploratory, descriptive and contextual study was conducted using in-depth unstructured face-to-face individual interviews at Untunjambili area under iLembe district in KwaZulu-Natal. A convenience sampling method was employed and participants aged between 18 and 49 years participated in the study.

**Results:**

Factors that hinder effective uptake of medical male circumcision are organised into five categories: (1) fear (fear of pain, fear of death, fear of HIV test and fear of delayed wound healing, (2) personal factors (age at circumcision, lack of role models, procrastination and lack of volition, fertility and faithfulness), (3) knowledge deficit on circumcision, (4) influence of culture and (5) natural circumcision.

**Conclusion:**

Factors that hinder effective uptake of medical male circumcision services are manifold. Cultivating a positive attitude towards medical male circumcision can promote uptake of circumcision services and a supportive social support system of men needs to be utilised to encourage men to be circumcised.

## Introduction

Circumcision is the surgical removal of the foreskin (prepuce) from the penis and the practice is widely performed on boys and young men in Africa and other parts of the world (Mandova,Mutonhori & Mudzanire 2013). Morris et al. (2016) explain that the estimated global prevalence of male circumcision is 38.7% and most of circumcised men are Jewish and Muslim. The World Health Organization (WHO) proposed a framework that seeks to contribute to the achievement of Millennium Development Goal (MDG) 6, which aimed at ensuring that the world has halted the spread of human immunodeficiency virus (HIV) by 2015 (WHO 2012). This is also in line with WHO and Joint United Nations Programme on HIV/AIDS (UNAIDS) recommendations that medical male circumcision (MMC) should be used as an additional important HIV prevention strategy for HIV prevention, particularly in settings with high HIV prevalence and low levels of male circumcision (Kenya National AIDS and STI Control Programme 2011).

Although the KwaZulu-Natal (KZN) province, where the study was conducted, once practised circumcision as a rite of passage before the times of King Shaka Zulu, this practice was disturbed in the Zulu tradition in the 19th century by King Shaka Zulu himself who believed it robbed him of many warriors at a time (Friedman, Rose & Titus 2011). In KwaZulu-Natal province, the MMC programme was re-introduced in May 2010 after discussions between His Majesty, Zulu King, King Goodwill Zwelithini Zulu, the then Premier Dr Zweli Mkhize, the KZN Department of Health and traditional leaders, who took the decision to implement a male circumcision programme as a prevention strategy against HIV and AIDS in an HIV-stricken province.

The observation has been that only younger boys are participating in the MMC programme instead of sexually active men. In most cases, only boys as young as 12 years have been observed to be participating in the programme, while older men seem to be dodging circumcision. Untunjambili Hospital District Health Information System (DHIS) data for the period April 2013 reveal that 65 men between the ages of 15 and 49 years were circumcised, while 82 boys who were circumcised at the same period were between 4 months and 14 years of age, and the same picture continued in May 2013 where 35 men between the ages of 15 and 49 years were circumcised, while 33 boys who were circumcised at the same period were between 4 months and 14 years. Nationally, 57% of men are circumcised and 30% of them were circumcised by a health worker, while 27% were circumcised by a traditional practitioner, family member or a friend (Department of Health 2016).

This initiative has not gone unchallenged by Zulu men who still believe they do not need to perform circumcision as it has not been part of their culture for decades. This article aimed at understanding factors that hinder effective uptake of MMC at Untunjambili area under iLembe district in KwaZulu-Natal province.

## Methods

### Research design and setting

A qualitative research design that is explorative, descriptive and contextual in nature was used to gain an understanding of factors that hinder effective uptake of MMC at Untunjambili area under iLembe district in KwaZulu-Natal province. Untunjambili is a rural area and most of its population comprises the Zulu tribe and it is under the governance of Maphumulo local municipality. Unstructured individual interviews were conducted to offer men a chance to express themselves without any interruptions. Brink, Van der Walt and Van Rensburg (2012) define interviews as the method of data collection in which an interviewer obtains responses from the participants in a face-to-face encounter, through a telephone call or by electronic means.

### Population and sampling

The study population comprised uncircumcised men aged between 18 and 49 years in the community of Untunjambili area under iLembe district in KwaZulu-Natal. These are men who were at outpatient department (OPD) at Untunjambili Hospital. While potential participants were in OPD queue, they were identified and all those who were willing to participate were approached. After identifying the population, a convenience sampling approach was used to select eligible participants. This approach involves accidental selection of readily available participants (Polit & Beck 2012). Nine men participated in the study – this number was informed by data saturation method as described by Polit and Beck (2012). Data saturation was reached when the researcher felt that participants repeatedly gave more or less the same information and no new information emerged during the interviews.

### Ethical considerations

Because this study involved human subjects, ethical approval was obtained from the University of South Africa (ethical clearance reference number HSHDC/260/2013), KZN Department of Health (ethical clearance reference number HRKM 331/13) and iLembe District Office. Participation in the study was voluntary and the participants were made aware of it. The participants were informed that their personal details will be kept confidential and anonymity will be maintained by using codes instead of names. In addition, they were made aware that they could withdraw from the study at any time without penalty. Informed consent was sought from the participants and consent forms were signed by men who were willing to participate in this study.

Ethical clearance was obtained from KwaZulu-Natal Department of Health (Ethical clearance number: HRKM 331/13).

### Data collection

In-depth face-to-face unstructured individual interviews and field notes were used to collect data. The researcher who is a nurse by profession conducted the interviews in a private space at Untunjambili Hospital OPD. Each interview took approximately 30–60 min and an audio recorder was used to record the interview for latter transcription. Two open-ended questions were asked and followed by more specific probing questions that depended on the answers obtained on the former questions. The participants were not confined to pre-specified response options, but were allowed to answer using their own words. To ensure that the research question is adequately addressed, the interviews were based on two central questions: ‘what are your views about medical male circumcision and why have you not taken up an opportunity to perform circumcision up to now?’ Facilitative communication skills as outlined by Uys and Middleton (2004) were used to help explore factors that hinder effective uptake of MMC services. Field notes on researcher’s perceptions and experiences were jotted down and kept as they assisted in gathering and interpreting data.

### Data analysis

Tech’s eight-step data analysis method was used to analyse the collected data (Creswell 2014). Data analysis in this article entailed categorising, ordering, manipulation and summarising of collected information. All transcripts were read carefully and similar topics were clustered together and the main themes were identified and described. The process of data analysis was conducted by the researcher and verified by an independent coder.

### Trustworthiness

For the research findings to be credible and used by the scientific community, the researchers utilised trustworthiness criteria as recommended by Lincoln and Guba (Streubert & Carpenter 2011). To ensure confidence in the truth of the data and interpretation of them, the researchers applied the credibility criterion. To ensure reliability of data after some time, the strategy of dependability was applied. To ensure objectivity, where the findings are the results and experiences of the respondents and not the preferences of the researchers, the strategy for confirmability was applied. To ensure the extent to which the findings of the study can be applicable in other settings or groups, a transferability criterion was considered (Polit & Beck 2012). In addition, the results are discussed with reference to the reviewed literature to compare them with the available literature.

## Results and discussion

The study results revealed that one participant was married, while eight were single. Out of the nine participants, one was a Further Education and Training student, three had passed grade 12 and there were five high school dropouts. In addition, three participants were employed, while six were unemployed. All the participants were African Zulu men who were in their 20s, 30s and 40s and were all uncircumcised. The findings are presented according to five major themes: (1) fear (fear of pain, fear of death, fear of HIV test and fear of delayed wound healing, (2) personal factors (age at circumcision, lack of role models, procrastination and lack of volition, fertility and faithfulness), (3) knowledge deficit on circumcision, (4) influence of culture and (5) natural circumcision.

### Theme 1: Fear

According to the interviewed men, fear is one of the factors that made them not to be circumcised. These men fear pain, death, HIV test and delayed wound healing.

#### Fear of pain

Some men were worried of pain that is caused by circumcision procedure. This fear of pain acts as a barrier for most men to be circumcised. Moabi and Mavundla (2018) explain that it is normal for men to experience pain after circumcision procedure as there has been a surgical incision. This is what some participants had to say in relation to fear of pain:

‘… [*A*]nd that we are afraid of, is pains …’ (P8, single, 32 years old)

‘Maybe since the part that is done circumcision is ‘the men’s home’, i.e. penis. This part is sensitive and the issue of feeling pains needs to be addressed.’ (P5, married, 42 years old)

‘… If males experience a drop (penile discharge), they complain of pains and they imagine that if they are cut in their penises, they think pain will be worse than that from drop (penile discharge).’ (P9, single, 33 years old)

The majority of studies conducted previously also identified pain as a major factor prohibiting men and boys from undergoing circumcision. Fear of pain during and after MMC surgical procedure is reported to be the major barrier for acceptability of circumcision (Scott, Weiss & Viljoen 2005). Fear of pain is not only a concern in South Africa, as Moabi and Mavundla (2018) explain, even in Lesotho, herd boys do not want to be circumcised because of fear of pain.

#### Fear of death

Fear of death is voiced out by some participants as one of the constraints to MMC. Some men think that death can be caused by cutting of some ligaments on the penis. This is what some participants had to say in relation to fear of death:

‘… We as boys are afraid that a certain ligament can be cut in the penis and you can end up dying and …’ (P6, single, 42 years old)

‘I may be unlucky and be called to die.’ (P9, single, 33 years old)

Mavundla et al. (2009) explain that during circumcision seasons, there is an increase in morbidity and mortality rates among young men and boys. Fear of death seems to be one of the constraints to circumcision, as Moabi and Mavundla (2018) explain that some men do not want to be circumcised because they believe that they can die while on the operation bed or at home after the procedure. Death after the procedure is believed to be caused by suturing the wound which was supposed to be let bleeding. Moabi and Mavundla (2018) contend that surgical wounds need to be sutured to prevent post-operative bleeding.

#### Fear of HIV test

Before every male is circumcised, he has to be tested for HIV. Some men are reluctant to find out their HIV status; hence, they refuse to be circumcised because of a precondition for circumcision, which is HIV testing. This is what some participants said about fear of HIV test:

‘… [*M*]ay be that has an impact but testing for HIV is also important and I do not know what to do to make people understand it better.’ (P1, single, 27 years old)

‘… [*E*]ven with the issue of HIV testing it was very difficult for me when I first tested but I had no choice since I was unwell. It was not easy…’ (P3, single, 32 years old)

Hartzol et al. (2014) explain that fear of HIV test is the major barrier to male circumcision uptake because men are required to first undertake HIV test. Moabi and Mavundla (2018) explain that HIV is the major issue in circumcision. They contend that some of their study participants refused to be circumcised because circumcision is not performed on HIV-positive men. This implies that before one is circumcised, he has to be tested for HIV and if HIV-positive, he is contraindicated to be circumcised.

#### Fear of delayed wound healing

Delayed wound healing tends to be in the mindset of some men and this acts as a barrier for effective uptake of MMC services. Some men believe that circumcision wound takes long to heal and that will affect their social life. Occupation can also predispose one to delayed wound healing, as described by some study participants. This is what they said in relation to delayed wound healing:

‘… [*A*]nd at times it takes longer to heal …’ (P1, single, 27 years old)

‘You find that you have long been done but it does not heal.’ (P3, single, 32 years old)

… I have heard young boys saying it takes longer to heal … (P7, single, 23 years old)

‘I have been driving tractors so I thought it was going to delay my wound healing since tractors can disturb the circumcised area …’ (P9, single, 33 years old)

Peltzer et al. (2008) contend that improving the quality of male circumcision services could reduce healing times and thus reduce the risk of HIV infection in those who resume sexual activity soon after circumcision. According to Herman-Roloff et al. (2011), delayed wound healing and prolonged time away from work are common barriers to the uptake of circumcision services among men.

Some seasons of the year can predispose one to delayed wound healing. This is according to some of the study participants who explained that they prefer to be circumcised in winter because wounds heal faster in winter. This is what one participant said:

‘I was thinking of doing it but I thought it could be better if I did it during winter months since my wounds heal faster in winter. I need to do it since I have a problem when I penetrate during sex, I experience cracks in my penis. I will consider it during winter months.’ (P4, single, 30 years old)

Moabi and Mavundla (2018) explain that even Basotho men want to be circumcised in winter because they believe that their wounds will heal faster.

### Theme 2: Personal factors

*Collins Concise Dictionary* (2004) defines ‘personal’ as belonging to or intended for a particular person and no one else. Some of these personal factors that were mentioned by participants included age at circumcision, lack of role models, procrastination and lack of volition, fertility and faithfulness.

#### Age at circumcision

Age at circumcision was seen by most men as a deterrent to performing circumcision because an impression has been created in the area of Untunjambili that only younger boys must be circumcised. There was also a general concern among the participants about the approach used by Untunjambili Hospital in recruiting men for circumcision. This is what some participants had to say:

‘… Especially the health workers, I think they only target mostly younger ages and they do not show the importance of circumcising in all age groups like ourselves in the 40s to encourage us. They say they want boys and not males to go for circumcision …’ **(**P9, single, 33 years old)

‘… No. I thought I must not interfere with the programme for the kids. I thought older ones are not acceptable because I saw the bus was coming to collect only school kids …’ (P8, single, 32 years old)

This is consistent with the studies conducted by Gasasira et al. (2012), who explain that some men believe that circumcision is for young and sexually promiscuous men. Moabi and Mavundla (2018) explain that circumcision can be done during neonatal period, childhood, adolescence and adulthood.

#### Lack of role models

*Collins Concise Dictionary* (2004) defines ‘role model’ as a person regarded by others, especially younger people, as a good example to follow. This factor was mentioned by participants that they are not circumcised because no one in their homes is circumcised. This is what they had to say in this regard:

‘… [*A*]nd at home my brother was against it because he read about it. At home as we speak there is no one who is circumcised.’ (P2, single, 35 years old)

This was congruent with a study conducted in KwaZulu-Natal on barriers and facilitators of male circumcision that uncovered that service providers targeting adolescent boys should not underestimate the importance of the involvement of families, intimate partners, peers and the community in general in a young man’s decision to undergo circumcision (George et al. 2014). This study revealed that immediate families have a significant influence on a young man’s decision, as boys are largely still in the care of parents or guardians. Siblings, especially brothers, were also found to share experiences and views about the importance of MMC.

#### Procrastination and lack of volition

*Collins Concise Dictionary* (2004) defines ‘procrastination’ as an act of delaying an action that must be done until later, often because it is unpleasant or boring, while volition is the act of exercising will power or cognitive process by which an individual decides on and commits to a particular course. As they say ‘procrastination is the thief of time’, different participants concurred with that. When asked why they are not circumcised up to now, some participants had this to say:

‘… The honourable one. I will say there is procrastination due to life issues and other problems in life …’ (P6, single, 42 years old)

‘… I had already made arrangements to go for circumcision but I got held up at work since I was relocated to work on another site. I need circumcision. I do not have a problem …’ (P1, single, 27 years old)

Pierotti and Thornton (2012) confirmed that opting to get circumcised in an adult male is a big decision, even with the potential benefits of the surgery. The period of inaction is explained as taking people’s time to accept a new practice. Once men have decided to undergo circumcision, no time should be wasted before circumcision is performed as they might change their minds later.

#### Fertility

*Collins Concise Dictionary* (2004) defines ‘fertility’ as the natural capacity to produce offspring. This means that others admire that they can get kids even if they are not circumcised. This factor was mentioned as a hindrance to the uptake of medical circumcision by some participants when asked why men are not considering to be circumcised. The following is a quote from one participant:

‘The older ones are saying they are able to get children even if they are not circumcised …’ (P6, single, 42 years old)

Sexual functioning and circumcision are related. This is according to Fink, Carson and Devellis (2002) who debate that circumcision reduces erectile function. They believe that a circumcised penis cannot erect to its full capacity. This is contrary to the study conducted by Moabi and Mavundla (2018) who explain that some men had a belief that circumcision promotes full penile erection because the foreskin which constricts the penis has been removed.

#### Faithfulness

Some men believe that there is no need for them to undergo circumcision, more especially if they remain faithful to their partners as there is no risk of sexual infections. These men believe that circumcision is only for men who are not faithful to their sexual partners. This is the direct quotation from one of the participants:

‘… I am married and I saw no need since I am faithful to my wife and she is faithful to me, and most boys do it because they are not faithful …’ (P1, single, 27 years old)

Hartzol et al. (2014) mention that older men in married relationships reported that they did not want to be circumcised because they were at low risk of HIV infection, while Pierotti and Thornton (2012) expanded this by stating that remaining faithful to one’s partner is also not seen as adequate protection from HIV because many men feel unable to trust their partners.

### Theme 3: Knowledge deficit regarding circumcision

Lack of knowledge regarding circumcision seems to be one of the factors that prevents effective uptake of circumcision services. Some men explained that there is a need to inform men and the public about circumcision and its importance. This is what some participants said in relation to lack of knowledge:

‘… May be they can try to bring together parents and their children so that they can all learn why circumcision is so important so that parents will know and understand.’ (P5, married, 43 years old)

‘… May be also the issue of awareness as you know most men do not visit a lot in areas like this …’ (P9, single, 33 years old)

Moabi and Mavundla (2018) explain that some of Basotho men are still having knowledge deficit with regard to the importance of circumcision. This is supported by Mugwanya et al. (2010) who explain that some men do not have knowledge about protective effects of circumcision.

### Theme 4: Influence of culture

Circumcision is perceived to be the practice of other cultures, not the Zulus. This is because during the time of King Shaka Zulu, circumcision was abolished and it was only done by few tribes, which includes Sothos and Xhosas. This is what some participants had to say in relation to culture:

‘When I was growing up as I was born in the 70s, it was not in the culture of the Zulus to circumcise.’ (P7, single, 23 years old)

‘It is not our culture to circumcise as Zulus. It is a Xhosa culture.’ (P6, single, 42 years old)

Most Zulus still believe that it is not in their culture to circumcise because they relinquished it in the 19th century during the times of King Shaka Zulu who believed it robbed him of many warriors at a time (Friedman et al. 2011). Moabi and Mavundla (2018) explain that some men regard circumcision as a tradition of other tribes and if they get circumcised, this implies that they are leaving their tradition to the different one.

### Theme 5: Natural circumcision

Natural circumcision refers to a phenomenon where the foreskin does not fully cover the glans penis (Community Baby Center 2014). It is believed that if the foreskin does not fully cover the glans penis, there is no need to undergo medical circumcision. This is what some participants had to say in relation to natural circumcision:

‘I think it is not all people who have long foreskin. Like myself, I have a very short foreskin, but …’ (P1, single, 27 years old)

‘I will say it (glans penis) is half exposed and I think something was going to be excised but I had that feeling that I am safer. Others are born with longer foreskin.’ (P6, single, 42 years old)

‘… Others think they are circumcised when they are not circumcised at all like me.’ (P2, single, 35 years old)

The *Mail & Guardian* explains that, with partial circumcision, the foreskin is not removed, but an elastic band of tissue under the penis glans is cut, allowing the foreskin to move easily back and forth and this allows sperm to move freely and enhances pleasure for men and women (Mail and Guardian Online 2009).

## Limitations

Quality of data in this study may have been compromised because of the sampling technique used. Uncircumcised men may have been under-represented or over-represented because readily available men were selected to participate in the study. For this matter, the results of the study may not be generalised to the whole Zulu nation as the findings are contextualised to the region where the study was conducted, that is, Untunjambili area.

## Recommendations

The following are recommendations for healthcare practice and research.

### Healthcare practice

Addressing fear of pain will bring about reassurance that the MMC procedure will not be unbearably painful, strengthening the ‘Hlolamanje − know your status’ campaign which will also encourage boys and men to know their HIV status well in advance.

In-depth community awareness about MMC must be provided. During interviews, it was clear that most men are totally against circumcision; this does not mean that they are ready for it; they still need to be motivated to correct their misconceptions about MMC. Not only men need to be provided with in-depth information in this regard, but also the community at large, *inter alia*, parents and women in particular, who may be partners to them or mothers or family heads.

### Research

There is a need to involve women in future studies as they are partners to men. The researchers believe that if women are involved, this could provide a more holistic sense of perceptions of how people in general feel about MMC, more especially a feminine perspective to circumcision.

## Conclusion

There are multiple factors that act as constraints to MMC. Fear is voiced by most of the participants as a constraint to seek circumcision services. There is a need to work hand-in-hand with support system of men to conquer these constraints. The findings of this study may be used by healthcare policy-makers to devise ways in which men can be motivated for circumcision.
